# In Situ Biosynthesis of Photothermal Parasite for Fluorescence Imaging-Guided Photothermal Therapy of Tumors

**DOI:** 10.3390/gels8110754

**Published:** 2022-11-21

**Authors:** Yaqiong Wang, Haiyan Pan, Zhaowei Meng, Cai Zhang

**Affiliations:** 1 Department of Radiology, Zhongshan Hospital, Fudan University, Shanghai 200032, China; 2 Department of Radiology, Tianjin Medical University General Hospital, Tianjin 300052, China; 3 Department of Nuclear Medicine, Tianjin Medical University General Hospital, Tianjin 300052, China; 4 Department of Radiology, Tianjin Medical University Cancer Institute and Hospital, Tianjin 300060, China

**Keywords:** alginate, hydrogel, genipin, biosynthesis, photothermal therapy

## Abstract

Photothermal therapy (PTT) has been widely known as a promising therapeutic strategy for cancer treatment in recent decades. However, some organic and inorganic photothermal agents exhibit shortcomings including potential long-term toxicity and lack of biodegradability. Biocompatible extracts from plants and animals provide several alternatives for the reformation of photothermal agents. Bio-inspired products still have inherent problems such as low accumulation in tumors, easy diffusion, and fast elimination. Herein, we aim to develop a biocompatible photothermal agent with tumor enrichment. Enlightened by “parasitized snails”, in situ biosynthesis of photothermal agents and fluorescence imaging-guided PTT are achieved with the assistance of alginate–calcium–genipin (ACG) hydrogel. ACG hydrogel is a mixture of alginate (ALG), calcium (Ca), and genipin (GP). Given that the crosslinking product of GP and protein displays fluorescent/photothermal features, the constructed ACG hydrogel can gradually react with the tumor and then “light up” and “ignite” the tumor under specific light excitation. The ACG hydrogel can be seen as a photothermal parasite, eventually leading to the death of tumor. The photothermal therapeutic effects of ACG hydrogel reacting with tumors are successfully proven in vivo. The naturally derived GP and ALG ensure the biosafety of the ACG hydrogel-based bio-application. This work is another successful practice of nature-inspired methodological strategy for in situ biosynthesis of the photothermal agent.

## 1. Introduction

Photothermal therapy (PTT) has emerged as an alternative method for tumor treatment in recent years due to its high selectivity and low invasiveness. The widely studied photothermal agents could improve the efficiency of PTT due to their higher accumulation in tumors than surrounding tissues and brilliant conversion ability of light energy into heat [1,2]. A variety of photothermal agents have been developed such as carbon nanostructures [3,4,5], noble metal nanomaterials [6,7,8,9], metal oxides and chalcogenides [10,11], semimetal nanoparticles [12,13], black phosphorus nanomaterials [14,15], photosensitizer-containing nanoparticles [16,17], and organic nanomaterials [18,19]. These photothermal agents possess outstanding absorption features and excellent therapeutic effects, while some lack biodegradability and potential biotoxicity hindering their further clinical translation tremendously.

Extracts from plants and animals provide several alternatives for the reformation of photothermal agents. Squid ink [20,21], bamboo charcoal [22,23], fruit-extracted anthocyanins [24,25], and black sesame [26,27] have been used to prepare photothermal agents which show brilliant biocompatibility. However, these bio-inspired photothermal agents exhibit inherent problems such as low accumulation in tumors, easy diffusion and fast elimination, which may cause side effects. A way to develop a biocompatible photothermal agent with the ability of specifically precise tumor enrichment is an urgent problem to be solved. 

Nature with intelligence can always provide us with answers to various questions. Terrestrial snails are likely to be parasitized by a species of double-disc trematode named leucochloridium paradoxum. The parasite can invade the snail’s tentacles, resulting in a weird, swollen, and pulsating appearance. The infected snail moves farther and higher until exposing itself at the top of the plant with better illumination. These appearance and behavior changes increase the visibility and accessibility of “zombie snails” to their predator birds, eventually resulting in the death of snails [28,29]. Inspired by this parasitic process, we aimed to generate a photothermal agent in situ in the tumor by supposing that a “photothermal parasite” seeded into the tumor. The “photothermal parasite” eventually makes the NIR laser prone to precisely exert photothermal ablation towards the tumor. 

**Scheme 1:**
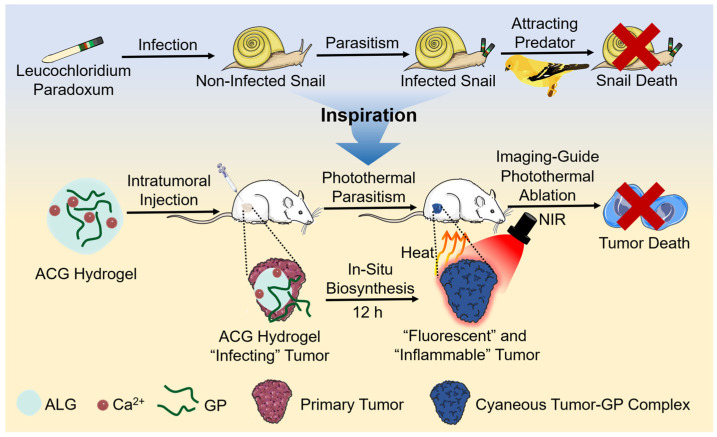
The schematic illustration of “photothermal parasite” practice: ACG hydrogel-assisted in situ biosynthesis of the tumor–GP complex for fluorescence imaging-guided PTT.

Genipin (GP) is first extracted from Chinese traditional medicine Gardenia fruits [30], the safety of which has been testified by forensic chemistry [31], food sciences [32], and cytology [33,34]. As a water soluble bifunctional crosslinking agent, GP could react with amine-containing molecules such as proteins in the presence of oxygen to generate gardenia blue, a fluorescent blue pigment [35,36]. Two reactions proceeding at diverse speeds lead to crosslinking between GP and primary amine groups. The quickest reaction is a nucleophilic attack on GP by primary amine groups, forming a heterocyclic compound of GP binding to basic protein residues. The second, slower reaction is the nucleophilic substitution of ester groups dominated by GP, forming a secondary amide linkage with protein [37]. The obtained dark blue protein–GP complex has a broad light absorption from ultraviolet (UV) to near-infrared (NIR) region, making it a promising candidate as a photothermal therapy agent [38,39]. Our previous work has proved that the fluorescent blue bovine serum albumin (BSA)–GP complex has a specific light absorption in the NIR region and could be applied as an excellent photothermal agent [40]. Theoretically, GP possesses the ability to react with the opulent oncoprotein, collagen, glycoprotein and proteoglycan in tumors, resulting in a dark-blue appearance of the tumor [35,41,42]. However, GP merely possesses 1% water solubility at room temperature (25 °C) [43,44], whose limited solubility hinders its application in vitro and in vivo. Although GP can be well dissolved in DMSO, the direct use of GP/DMSO solution is unbearable with great biosafety concerns as the definite carcinogenicity of DMSO. Alginate–calcium (ALG–Ca) hydrogel with strong disperse ability and good biocompatibility provides a feasible solution for the homodisperse of GP [45,46]. Herein, we proposed an in situ biosynthesis method by introducing a “photothermal parasite”-ALG-Ca-GP (ACG) hydrogel into the tumor. In vitro, the ACG hydrogel could react with BSA or cell medium, resulting in a bluish product with brilliant fluorescent and photothermal effect. In vivo, the ACG hydrogel-infected tumor gradually changed into a dark-blue look, and fluorescence imaging-guided localized PTT could be realized after 12 h “parasitism” (Scheme 1). Compared with other photothermal agents that are entirely synthesized in vitro [2,19,47], our designed ACG hydrogel can use the tumor itself as a “raw material” to generate “fuel”. On the one hand, it increases the additional consumption of tumor tissue, and on the other hand, it provides a more specific and biocompatible photothermal product for personalized PTT. This work, inspired by the parasitism phenomenon in nature, provided a novel methodological strategy for in situ biosynthesis of photothermal agents to accomplish localized PTT precisely.

## 2. Results and Discussion

### 2.1. Synthesis and Characterization of ACG Hydrogel

**Figure 1:**
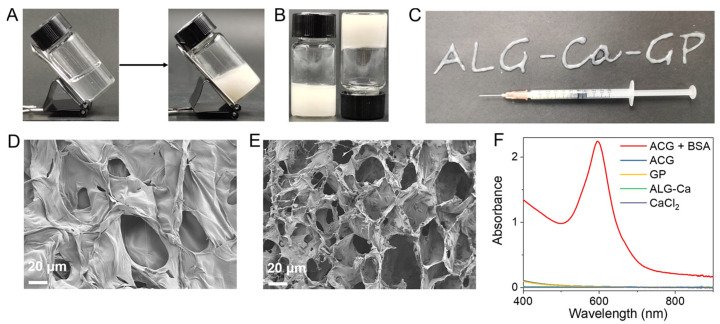
Characterization of the ACG hydrogel. (**A**) The tilted ALG–Ca hydrogel (left) and ACG hydrogel (right). (**B**) The photograph of ACG hydrogel (upright and inverted). (**C**) The pattern of “ALG–Ca–GP” is coherently formed by the ACG hydrogel with a 1 mL syringe. SEM image of ALG–Ca hydrogel (**D**) and ACG hydrogel (**E**), Scale bar: 20 μm. (**F**) UV–vis–NIR absorption spectra of ACG + BSA (10 mg/mL) hydrogel, ACG hydrogel, ALG–Ca hydrogel, GP and CaCl_2_ after fourfold dilution.

The ACG hydrogel was synthesized by mixing ALG, CaCl_2_, and GP at room temperature. As a natural polysaccharide, ALG solution could quickly convert to hydrogel in an ultrasimple way once exposed to multivalent cations (such as Ca^2+^ and Mg^2+^) [48]. Different concentrations of ALG solution and CaCl_2_ solution were optimized, as shown in the Supplementary Figure S1A–D, to obtain the optimal dosage of ALG and Ca^2+^ for the formation of hydrogel. Finally, ALG (5 mg/mL) and CaCl_2_ (0.5 mg/mL) were chosen as the optimum concentrations to obtain a transparent ALG–Ca hydrogel with good stability (Figure 1A). The ACG hydrogel was prepared by dropping the CaCl_2_ solution into the GP-dispersed ALG solution, and the concentration of GP was further optimized. Supplementary Figure S2A–D showed that the addition of GP with various concentrations (1–20 mg/mL) had no significant influence on the gelation process driven by ALG and Ca^2+^, and GP could uniformly disperse in the ACG hydrogel. When 25 mg/mL of GP was added into the system, the obtained hydrogel showed a degree of maldistribution and fragility. To balance the integrity of the hydrogel with its high loading capacity, the preparation method of ACG hydrogel was finally confirmed as a mixture of ALG (5 mg/mL), CaCl_2_ (0.5 mg/mL), and GP (20 mg/mL) together and stored for the following characterization and bio-application (Figure 1A).

To verify the strong loading ability of ALG–Ca hydrogel, we prepared GP solution and ACG hydrogel with the same GP concentration (20 mg/mL) which was left to stay still for 12 h. The ACG hydrogel kept a homogeneous distribution state, but apparent precipitation was found in the GP solution after 12 h of immobility (Supplementary Figure S3). The optimized ACG hydrogel was capable of maintaining its original shape regardless of being tilted or inverted (Figure 1A,B). Owing to its easy injectability, ACG hydrogel could draw specific patterns via a 1 mL syringe (Figure 1C). Rheological experiments showed that the storage moduli (G′) of ALG-Ca^2+^ hydrogel, ACG hydrogel, and ACG + BSA hydrogel were higher than their loss moduli (G″), which demonstrated their gel state (Supplementary Figure S4). In addition, the G′ of ALG-Ca^2+^ hydrogel, ACG hydrogel, and ACG + BSA hydrogel decreased and were lower than its G″ as the shear rate increased, indicating their injectable state (Supplementary Figure S5). The scanning electron microscopy (SEM) image confirmed the smooth, porous network structure of ALG–Ca hydrogel (Figure 1D), while for ACG hydrogel, abundant GP granules were found evenly interspersing in the porous network scaffold of hydrogel (Figure 1E). The UV–vis–NIR absorption spectrum of the ACG hydrogel further demonstrated the successful loading of GP to the ALG–Ca hydrogel (Figure 1F).

### 2.2. In Vitro Evaluation of ACG Hydrogel in a Simulated Protein-Rich Environment

**Figure 2:**
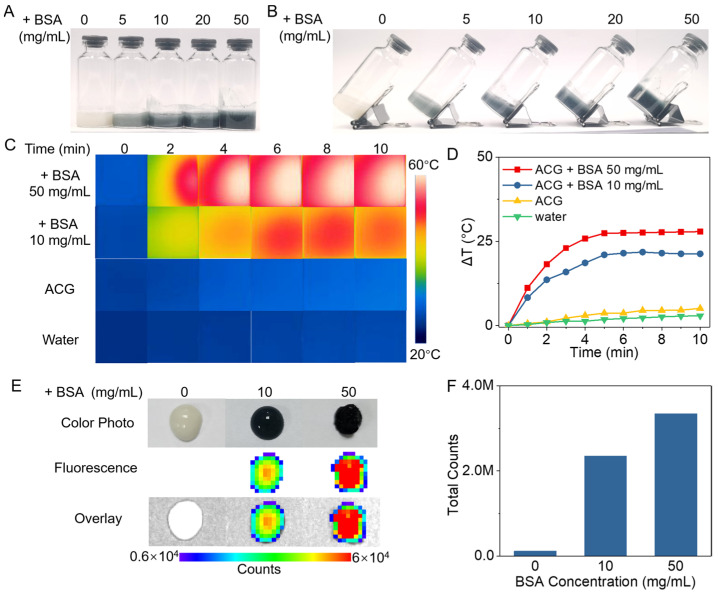
In vitro evaluation of the properties of ACG hydrogel in a simulated protein-rich environment. Digital photos of the upright (**A**) and tilted (**B**) ACG + BSA hydrogel with different concentrations of BSA (0, 5, 10, 20, 50 mg/mL). Infrared thermal photos (**C**) and photothermal heating curves (**D**) of ACG + BSA hydrogel (0, 10, and 50 mg BSA/mL) and water under 808 nm laser irradiation (2 W/cm^2^, 10 min). The fluorescence imaging (**E**) and the fluorescent counts of the region of interest (ROI) (**F**) of ACG + BSA hydrogel (0, 10, and 50 mg BSA/mL).

It is well known that tumor tissue is a protein-rich environment. To pre-verify the potential of ACG hydrogel as a “photothermal parasite” in the tumor, BSA was used as the protein source to simulate a protein-rich tumor environment in vitro. Figure 2A,B showed a series of blue hydrogels acquired by incubating ACG hydrogel with different concentrations of BSA after 12 h. The UV–vis–NIR absorption spectrum of the ACG + BSA hydrogel illustrated a definite absorption peak at 596 nm wavelength, which has been proven as the characteristic peak for the successful crosslink of GP and protein, and the absorption of the ACG + BSA hydrogel at 808 nm still reached 0.2 after fourfold dilution compared with the corresponding absorption of ACG hydrogel at 0.01 (Figure 1F) [40]. In order to evaluate the photothermal effect of the ACG + BSA hydrogel, ACG + BSA hydrogel with various BSA concentrations (10 and 50 mg BSA/mL) was irradiated by an 808 nm laser (2 W/cm^2^, 10 min), the pure water and ACG hydrogel were set as control. The infrared thermal photos in Figure 2C showed that the temperature of ACG + BSA hydrogel significantly increased during the irradiation, and the ACG + BSA hydrogel intuitively displayed a brilliant photothermal temperature elevation in a BSA concentration-dependent manner. Figure 2D showed the temperature of ACG + BSA hydrogel at least raised by 21.3 °C, and the temperature of ACG + BSA hydrogel containing 50 mg/mL of BSA even increased by 27.9 °C, while pure water and ACG hydrogel only showed a slight temperature rise of 2.9 °C and 5.1 °C, respectively. Moreover, the fluorescent spectra of ACG + BSA hydrogel showed a maximum fluorescent emission at ~616 nm with a 550 nm excitation (Supplementary Figure S6) [49], and the fluorescence imaging of ACG + BSA hydrogels also illuminated their bright fluorescence after 12 h of incubation (Figure 2E,F).

### 2.3. Cytotoxicity of ACG Hydrogel

The cytotoxicity of ACG hydrogel was evaluated via the standard MTT assay. ACG hydrogel with different concentrations (0 to 150 μg GP/mL) was incubated with 4T1 cells for 12 h. The results in Supplementary Figure S7 showed negligible cell death after the incubation. High cell viability (over 80%) demonstrated the low cytotoxicity of ACG hydrogel.

### 2.4. In Vitro Evaluation of PTT Effect of GP-Based Complex

**Figure 3:**
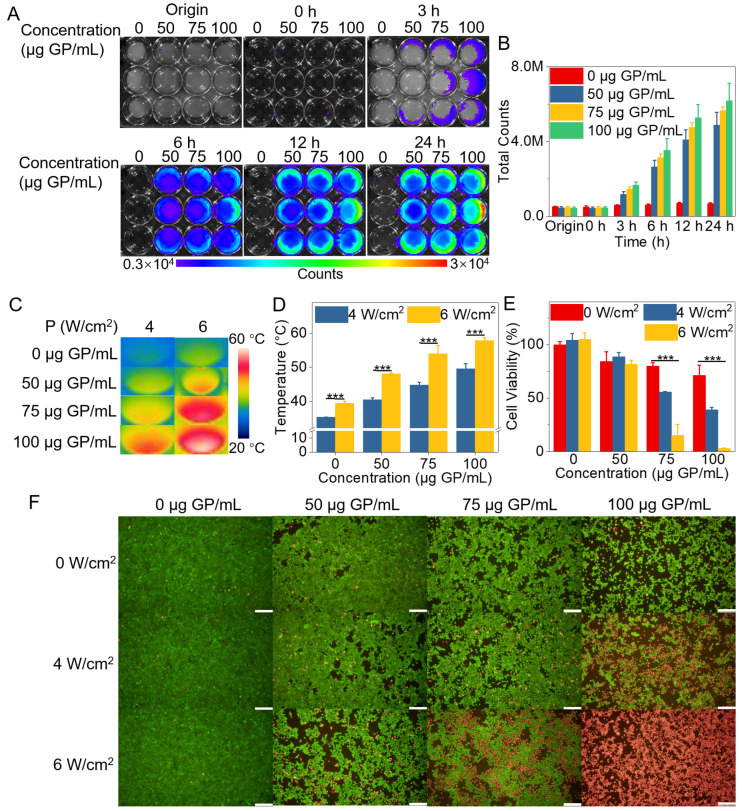
The cytological experiment of ACG hydrogel in vitro. (**A**) The fluorescent images of the 4T1 cells incubated with ACG hydrogel (0, 50, 75, and 100 μg GP/mL) for 0 h, 3 h, 6 h, 12 h, and 24 h, *n* = 3. (**B**) The quantitative analysis of fluorescent counts in each well of (**A**), shown as means ± SD, *n* = 3. (**C**) The infrared thermal photos of 4T1 cells incubated with ACG hydrogel (0, 50, 75, and 100 μg GP/mL) under 808 nm laser irradiation (4 or 6 W/cm^2^, 10 min). (**D**) The quantitative analysis of temperature change of different treatments, shown as means ± SD, *n* = 3, was evaluated by two-way ANOVA tests, *** *p* < 0.001. (**E**) Cell viabilities of 4T1 cells incubated with ACG hydrogel (0, 50, 75, and 100 μg GP/mL) under 808 nm laser irradiation (0, 4, and 6 W/cm^2^, 10 min), shown as means ± SD, evaluated by two-way ANOVA tests, *** *p* < 0.001. (**F**) The dual staining of living/dead cells with Calcein AM and PI is shown as green and red, respectively. Scale bar: 200 μm.

Given that gardenia blue can be produced once GP reacts with amine-containing molecules in the presence of oxygen [35,36], it is theoretically possible for GP to react with the culture medium containing glutamine and fetal bovine serum [50,51], since these components are abundant in amine groups. An appropriate incubation time of the “photothermal parasite” within the cell environment was a crucial factor for generating photothermal substances. Thus, we optimized the incubation time of ACG hydrogel with the 4T1 cells. The culture medium incubated with ACG hydrogel gradually turned red to purple and showed a fluorescence signal at 3 h. The fluorescence intensity of the mixture significantly enhanced with the extension of incubation time. A sufficient fluorescent signal of the mixture was achieved when the ACG hydrogel was incubated with the cell medium for 12 h, and the color turned dark purple (Figure 3A,B), demonstrating that the GP-based complex was generated. Thus, ACG hydrogel-assisted in vitro PTT was performed after 12 h incubation with cells. After being irradiated by an 808 nm laser (4 or 6 W/cm^2^, 10 min), the cell samples cultured with ACG hydrogel showed a concentration-dependent temperature rise with statistical significance. The cell temperature of the cell samples treated with 100 μg GP/mL of ACG hydrogel and 6 W/cm^2^ laser irradiation even increased to 57.9 °C, which provided sufficient temperature for the thermal ablation of 4T1 cells (Figure 3C,D). MTT assay reflected the cell viability of the cell samples treated with ACG hydrogel (75 and 100 μg GP/mL) and different power densities of 808 nm laser irradiation possessed evident decrease with statistical differences. The cell viability even decreased to 3% when the ACG hydrogel concentration reached 100 μg GP/mL and the laser irradiation power up to 6 W/cm^2^ (Figure 3E). In contrast, the cell samples treated with ACG hydrogel or laser irradiation alone showed negligible cell death. Moreover, living/dead cell dual staining differentiated the living (shown in green) and dead cells (shown in red) after different treatments (Figure 3F). The above results proved the remarkable photothermal efficacy of the GP-based complex generated by the ACG hydrogel and the tumor cell environment in vitro.

### 2.5. Time Optimization of Intratumoral Injection of ACG Hydrogel in Mice

**Figure 4:**
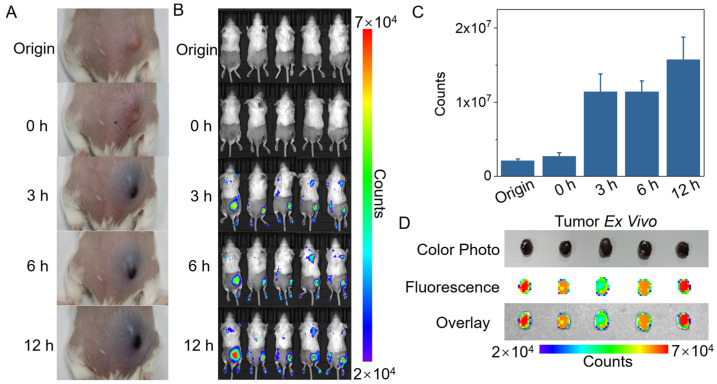
Time optimization of intratumoral injection of ACG hydrogel in mice. (**A**) Digital photographs of tumor color change derived from the reaction of ACG hydrogel with tumor during 12 h. (**B**) In vivo imaging of tumor fluorescence signal change derived from the reaction of ACG hydrogel with tumor during 12 h. (**C**) The quantitative analysis of fluorescent counts of tumor sites in (**B**) is shown as means ± SD, *n* = 5. (**D**) Blue tumors exfoliated at 12 h after intratumoral injection of ACG hydrogel. Up: color digital photograph. Middle: fluorescence photo. Down: overlay of grey photo and fluorescence photo.

Similar to the cellular experiment, proper “parasitical” time was undoubtedly crucial for the “photothermal parasite” to convert a “noninflammable” tumor to an “inflammable” one. Solid tumor tissue can be divided into tumor parenchyma and tumor stroma. The tumor parenchyma with tissue specificity contains a large number of tumor cells, which composes the main component of the tumor. The tumor stroma without tissue specificity is composed of connective tissue, blood vessels, and lymphatic vessels, which mainly support and nourish tumor cells. Tumor parenchyma and stroma are both rich in various protein components, including oncoprotein, collagen, glycoprotein and proteoglycan [41,42]. Thus, GP can react with these amine-containing protein components in tumors to produce blue substances [35,36]. The tumor-bearing mice were intratumorally injected with 50 μL of ACG hydrogel, and the tumor appearance and fluorescent signal changes were monitored at 0 h, 3 h, 6 h, and 12 h post-injection. Definite blue color and fluorescent signal were observed in the tumor site at 3 h post-injection, and the blue color of tumor gradually became darker, accompanied by a stronger fluorescent signal in 12 h (Figure 4A–C). The resected tumor showed a blue appearance and bright fluorescent signal, which demonstrated the successful alteration of the tumor driven by “photothermal parasite”-ACG hydrogel within 12 h (Figure 4D).

### 2.6. In Vivo PTT Assisted by the “Parasitism” of ACG Hydrogel in Tumor

**Figure 5:**
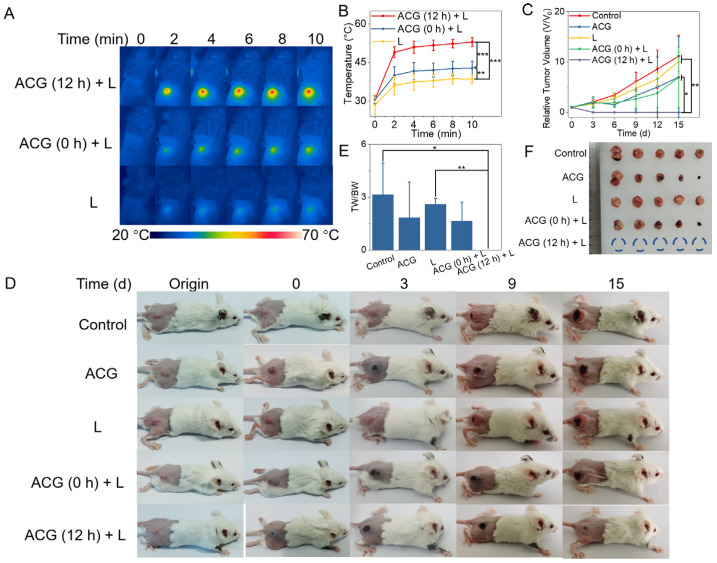
In vivo PTT assisted by the “parasitism” of ACG hydrogel in the tumor. (**A**) Infrared thermal photos of tumor-bearing mice during 808 nm laser irradiation, *n* = 5. (**B**) The average temperature change curves of mice’s tumor surface during 808 nm laser irradiation, shown as means ± SD, complied with the normal distribution and evaluated by one-way ANOVA tests, ** *p* < 0.01, *** *p* < 0.001. (**C**) The average relative tumor volume (V/V_0_) change of mice in each group, shown as means ± SD, complied with the normal distribution and evaluated by one-way ANOVA tests, * *p* < 0.05, ** *p* < 0.01. (**D**) The digital photographs of tumor-bearing mice at different time points (Origin, 0, 3, 9, and 15 d) in each group, *n* = 5. (**E**) The ratio of tumor weight to body weight of mice in each group, shown as means ± SD, not complied with the normal distribution and evaluated by nonparametric Kruskal–Wallis tests, * *p* < 0.05, ** *p* < 0.01. (**F**) Photo of tumor masses excised from tumor-bearing mice after different treatments. ACG: intratumoral injection of 50 μL ACG hydrogel. L: 808 nm laser irradiation (2 W/cm^2^, 10 min). ACG (0 h) + L: intratumoral injection of 50 μL ACG hydrogel followed by immediate laser irradiation (2 W/cm^2^, 10 min). ACG (12 h) + L: intratumoral injection of 50 μL ACG hydrogel followed by 12 h delayed laser irradiation (2 W/cm^2^, 10 min). TW: Tumor Weight. BW: Body Weight.

The tumor-bearing BALB/C mice were randomly divided into 5 groups (*n* = 5) for different treatments to investigate the feasibility of the “photothermal parasite” of ACG hydrogel. According to the fluorescence imaging results, a significant fluorescent signal of the tumor was observed at 12 h post-injection, which revealed that the ACG hydrogel had fully reacted with endogenous tumor molecules. Therefore, the optimal therapeutic time for in vivo PTT was determined to be 12 h after intratumoral injection to obtain enough photothermal effect and less distribution of surrounding tissues. Due to the lack of enough time for the production of tumor-GP complex in situ, ACG (0 h) + L group with laser irradiation immediately after injection of ACG hydrogel did not reveal an ideal temperature rise, and the temperature of the tumor in L group only increased to 38.5 °C. In comparison, the tumor displayed a bluish appearance after 12 h “parasitism” of ACG hydrogel, which indicated a sufficient production of photothermal substances in the tumor. As expected, the ACG (12 h) + L group gave an impressive temperature rise to 52.9 °C on the tumor surface (Figure 5A,B). The average relative tumor volume (V/V_0_) change during 15 days manifested no tumor growth in ACG (12 h) + L group compared to other groups with significant differences (Figure 5C). The digital photographs of tumor-bearing mice at different time points (Origin, 0, 3, 9, and 15 d) intuitively confirmed that the tumors in ACG (12 h) + L group were successfully ablated and scarred, while the tumor kept growing in other groups (Figure 5D). The remarkable photothermal therapeutic effect of the GP-based complex generated from the ACG hydrogel and tumor was also verified by the ratio of tumor weight to body weight of the mice on the 15th day post-treatment (Figure 5E). The tumor was completely ablated in the ACG (12 h) + L group, and the exfoliated tumor from other groups exhibited different sizes (Figure 5F). It is worth noting that the ACG alone group and ACG (0 h) + L group displayed a similar tumor size, even with an inadequate photothermal effect, but just ACG hydrogel in the tumor site also led to a certain extent of tumor shrinkage. This phenomenon could be explained that the crosslink of GP might partially impair the expansive growth feature of tumor tissue since the protein denaturation is accompanied during the crosslinking process. A large number of tumor cells without evident necrosis were found in the representative H&E staining pictures of the tumor tissues in the control group, ACG alone group, L group, and ACG (0 h) + L group (Supplementary Figure S8). These results demonstrated that the ACG hydrogel could serve as an efficient “photothermal parasite” to in situ generate the GP-based complex in tumor tissue based on a protein-rich tumor environment and exert the splendid photothermal effect to kill tumors.

### 2.7. In Vivo Toxicity Assessment of ACG Hydrogel

**Figure 6:**
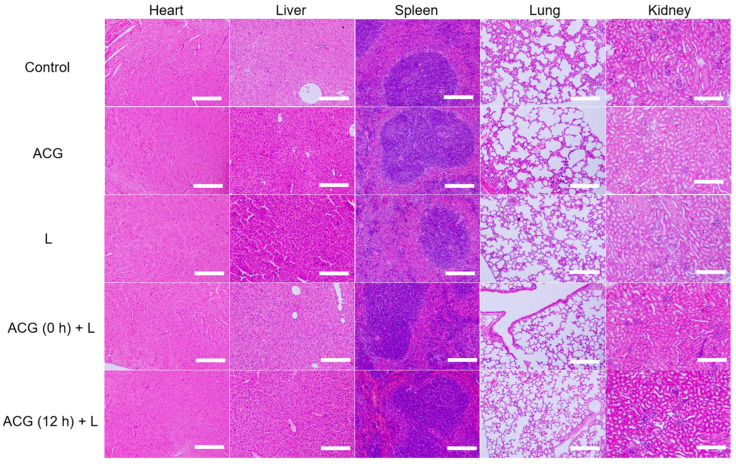
The representative hematoxylin and eosin (H&E) stained tissue sections of mice’s major organs, including heart, liver, spleen, lung, and kidney, after different treatments. Scale bar: 200 μm. ACG: intratumoral injection of 50 μL ACG hydrogel. L: 808 nm laser irradiation (2 W/cm^2^, 10 min). ACG (0 h) + L: intratumoral injection of 50 μL ACG hydrogel followed by immediate laser irradiation (2 W/cm^2^, 10 min). ACG (12 h) + L: intratumoral injection of 50 μL ACG hydrogel followed by 12 h delayed laser irradiation (2 W/cm^2^, 10 min).

**Figure 7:**
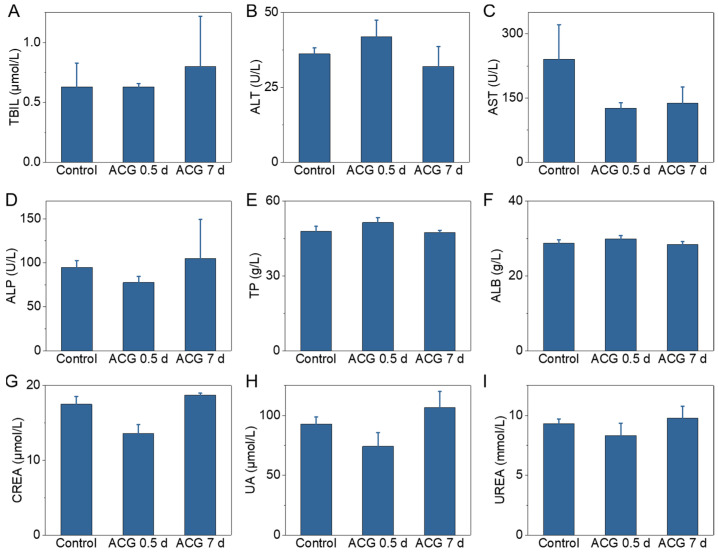
The biochemical analysis of mice with different treatments. (**A**) TBIL. (**B**) ALT. (**C**) AST. (**D**) ALP. (**E**) TP. (**F**) ALB. (**G**) CREA. (**H**) UA. (**I**) UERA. Data shown as means ± SD, *n* = 3. Control: blood sample collection of tumor-bearing mice without other interference. ACG 0.5 d: blood sample collection at 0.5 d after intratumoral injection of 50 μL ACG hydrogel. ACG 7 d: blood sample collection at 7 d after intratumoral injection of 50 μL ACG hydrogel.

After in situ tumor PTT experiment, the major organs (heart, liver, spleen, lung, and kidney) of the mouse in each group were collected and sliced for H&E staining for the toxicological evaluation. No apparent morphological change, inflammation, cell apoptosis or necrosis were found in mice’s main organs from different groups (control, ACG, L, ACG (0 h) + L, and ACG (12 h) + L) (Figure 6). Treatments did not cause abnormal body weight decrease in the mice, which demonstrated the excellent biosafety of the ACG hydrogel “parasitism” in tumors (Supplementary Figure S9). To fully understand the potential toxicity in vivo of ACG hydrogel, the short-term (0.5 d) and long-term (7 d) toxicity of mice with intratumoral injection of ACG hydrogel was assessed through serum biochemical analysis. The liver function indicators including TBIL, ALT, AST, ALP, TP, and ALB and the kidney function indicators including CREA, UA, and UREA were monitored. All the biochemical indicators of the mice with intratumoral injection of ACG hydrogel exhibited minor change compared to those without any treatment at 0.5 d and 7 d post-injection (Figure 7A–I). The above in vivo toxicity assessment of ACG hydrogel comprehensively demonstrated that the ACG hydrogel possessed negligible toxicity and excellent biocompatibility in vivo.

## 3. Conclusions

Inspired by “parasitized snails” in nature, we proposed a “photothermal parasite” method to implant the ACG hydrogel in the tumor to in situ fabricate the GP-based complex based on the released GP from ACG hydrogel and abundant proteins in the tumor environment. In this study, the “photothermal parasite”-ACG hydrogel was synthesized by simply mixing ALG, Ca, and GP at room temperature. The existence of Ca^2+^ could easily transform ALG–GP from a solution state to a hydrogel state, producing a uniform distribution of GP in ACG hydrogel. In a simulated protein-rich tumor environment in vitro, the ACG hydrogel gradually reacted with BSA and showed excellent fluorescence properties and photothermal effects within 12 h. The effectiveness of the in situ biosynthesis strategy in producing GP-based complex based on ACG hydrogel and serum protein, glutamine in the cell medium was also successfully testified at the cellular level. Furthermore, intratumoral injection of ACG hydrogel successfully enabled real-time optical bioimaging of the tumor, guiding the optimal time window for PTT. Complete tumor photothermal ablation was achieved by implantation of ACG hydrogel for 12 h and combination with 808 nm laser irradiation. In vitro and in vivo toxicity assessments indicated the good biocompatibility of ACG hydrogel owing to the inherent biosafety of GP and ALG. Compared with other photothermal agents that are entirely synthesized in vitro, our designed ACG hydrogel can use the tumor itself as a “raw material” to generate “fuel”. On the one hand, it increases the additional consumption of tumor tissue, and on the other hand, it provides a more specific and biocompatible photothermal product for personalized PTT. This work provided a novel methodological strategy for in situ biosynthesis of photothermal agents in tumors to accomplish imaging-guided localized PTT.

## 4. Materials and Methods

### 4.1. Reagents and Materials

The chemical materials were all at least of analytical grade. Genipin (98%) was bought from LinChuan ZhiXin Biotechnology Co., Ltd. (Jiangxi, China). Sodium alginate, CaCl_2_, and methyl thiazolyl tetrazolium (MTT) were purchased from Aladdin Reagent Co., Ltd. (Shanghai, China). Bovine serum albumin (BSA) was purchased from Beijing Dingguo Biotechnology Co., Ltd. (Beijing, China). Ethanol and dimethyl sulfoxide (DMSO) were purchased from Concord Technology (Tianjin, China). Paraformaldehyde was provided by Servicebio Biotechnology Co., Ltd. (Wuhan, China). Ultrapure water manufactured by Hangzhou Wahaha Group Co., Ltd. was used throughout the synthesis procedure. Calcein acetoxymethyl ester (Calcein AM) and propidium iodide (PI) were obtained from Dojindo Laboratories (Shanghai, China). Dulbecco’s Modified Eagle Medium (DMEM) and 0.25% Trypsin-EDTA were bought from Gibco Co. (Billings, MT, USA). Fetal bovine serum (FBS) was obtained from Excell Bio Biotechnology Co., Ltd. (Shanghai, China). Penicillin–Streptomycin solution was bought from Solarbio Science & Technology Co., Ltd. (Beijing, China).

### 4.2. Characterizations

The morphology of the prepared hydrogel was characterized by scanning electron microscopy (SEM) (Gemini 300-3ViewXP, Zeiss, Oberkochen, Germany). The UV–vis–NIR absorption spectra were determined by a UV-3600 plus spectrophotometer (Hitachi, Tokyo, Japan). Rheology experiments were conducted on a rheometer (MCR 302, Anton Paar, Graz, Austria). ALG (5 mg/mL), CaCl_2_ (0.5 mg/mL), GP (20 mg/mL) and BSA (10 mg/mL) were chosen as the fixed concentrations to investigate the rheological properties. The fluorescent spectra were recorded on an FL-4500 spectrofluorometer (Hitachi, Tokyo, Japan) equipped with a plotter unit and a quartz cell (1 cm × 1 cm). Fluorescent imaging was performed on an IVIS^®^ Lumina III In vivo Imaging System (PerkinElmer, Waltham, MA, USA). The Photothermal experiment was investigated with an 808 nm laser device produced by Hi-Tech Optoelectronics Co., Ltd. (Beijing, China). The infrared thermal photos were taken by an E50 series infrared thermometer camera (FLIR, Wilsonville, OR, USA).

### 4.3. Preparation of ACG Hydrogel

ACG hydrogel was synthesized by the Ca^2+^-induced cross-linking strategy. Briefly, 200 mg sodium alginate powder was dissolved in 10 mL water under magnetic stirring for 2 h at room temperature to obtain a homogeneous state. Then, ALG solution (20 mg/mL, 0.25 mL) was mixed with GP (20 mg) in a bottle under magnetic stirring, and then 0.7 mL water was supplemented. Afterward, the CaCl_2_ solution (10 mg/mL, 0.05 mL) was dropwise added into the GP-loaded ALG solution and kept stirring for 4 h to achieve an even distribution of GP. Then, 8 h setting was required to form the steady ACG hydrogel.

### 4.4. In Vitro Evaluation of GP-Based Complex

To evaluate the photothermal ability of the ACG hydrogel in a simulated protein-rich tumor environment, the BSA solutions with final concentrations of 0, 5, 10, 20, and 50 mg/mL were introduced to ACG hydrogel under magnetic stirring and kept stirring for 4 h, then incubated for 8 h for further characterization. Then, 1 mL of the ACG + BSA hydrogel with various concentrations of BSA (0, 10, and 50 mg BSA/mL) was respectively drawn into a quartz cuvette by a syringe and then exposed to 808 nm laser irradiation (2 W/cm^2^, 10 min). The temperature changes during laser irradiation were monitored by an infrared camera. The ACG + BSA hydrogels (0, 10, 50 mg BSA/mL) were drawn onto a black hardboard for fluorescent imaging with an excitation wavelength of 605 nm and an emission filter of Cy5.5. The fluorescent counts of the hydrogel drops were analyzed quantitatively. The UV–vis–NIR absorption and fluorescence emission spectra of ACG and ACG + BSA hydrogel were also explored.

### 4.5. In Vitro Cytotoxicity Assay

The cytotoxicity of ACG hydrogel was evaluated by the standard MTT test using 4T1 cells. The 4T1 cells were cultured in DMEM with 10% FBS and 1% streptomycin–penicillin in a constant atmosphere (5% CO_2_, 37 °C). The cells were seeded in a 96-well plate with 1 × 10^4^ per well and cultured for 24 h. Then, the cells were washed with phosphate buffer saline (PBS, 10 mM, pH = 7.4) to remove dead cells and incubated with the fresh medium that contained ACG hydrogel (0 to 150 μg GP/mL). After 12 h incubation, the cells were treated with MTT (10 μL, 5 mg/mL) and 190 μL fresh medium, and then incubated for 4 h in a CO_2_ incubator. Then, DMSO (120 μL) was separately added to each well to replace the MTT-contained medium and dissolve the purple formazan crystals in the bottom of each well. The absorbance at 490 nm of the cell samples was recorded after a 10 min shaking procedure by a microplate reader (Bio-Tek, Winooski, VT, USA). The cell viability was calculated according to the following formula:

Cell viability = OD_exp_/OD_con_ × 100%, (1)

where OD_exp_ and OD_con_ are the optical density (OD) of the cells treated with different concentrations of ACG hydrogel and the cells in the control group, respectively.

### 4.6. Optimization of Incubation Time of ACG Hydrogel and 4T1 Cells

To achieve satisfactory in vitro PTT outcomes of the GP-based complex produced by the ACG hydrogel and the serum protein and glutamine in the cell medium, we optimized the incubation time of ACG hydrogel with 4T1 cells. Various concentrations of ACG hydrogels (0, 50, 75, and 100 μg GP/mL) were incubated with 4T1 cells, during which the fluorescent images of ACG-incubated cells were collected at 0 h, 3 h, 6 h, 12 h, 24 h with a 605 nm excitation laser and an emission filter of Cy5.5. Finally, the fluorescent counts of cells were analyzed quantitatively.

### 4.7. Evaluation of In Vitro PTT Effect of GP-Based Complex

4T1 cells were seeded in a 96-well plate at a density of 1 × 10^5^ per well and cultured in a constant atmosphere (5% CO_2_, 37 °C) for 8 h. Then, the cells were incubated with different concentrations of ACG hydrogel (0, 50, 75, and 100 μg GP/mL) for 12 h, followed by 808 nm laser irradiation (0, 4, and 6 W/cm^2^, 10 min). The infrared thermal photos of the cell samples with different treatments were recorded by an infrared camera. The cell viabilities were studied by a standard MTT assay as aforementioned. Calcein AM and PI were used to stain living and dead cells after different treatments. After incubation for 10 min, each well was washed twice with PBS carefully to remove the free dyes. The living/dead cell dual-staining fluorescence images were then observed and collected by an inverted fluorescence microscope (IX73, Olympus, Tokyo, Japan).

### 4.8. Animal Model

All animal procedures were approved by the ethics committee of Tianjin Medical University Cancer Institute and Hospital (Approval Number: AE-2022061) and in accordance with National Institutes of Health Guidelines. The tumor model was built through subcutaneous injection of 1 × 10^6^ 4T1 cells on the right side of the back of BALB/c mice (15–20 g, HFK Bioscience Co., Ltd., Beijing, China). In vivo experiments were carried out when the tumor size reached 5–8 mm in diameter.

### 4.9. Optimization of Incubation Time of ACG Hydrogel and Tumor Tissues In Vivo

ACG hydrogel with a volume of 50 μL was intratumorally injected into the tumor-bearing mice (*n* = 5). Then, digital photos and fluorescence imaging of each mouse were simultaneously collected before and after injection 0 h, 3 h, 6 h, and 12 h to confirm the optimal incubation time of ACG hydrogel in the tumor tissue for further in vivo PTT. The tumor of each mouse was exfoliated at 12 h post-injection, and digital photos and fluorescence images of the tumors were also collected.

### 4.10. In Vivo PTT of the “Parasitism” of ACG Hydrogel in Tumor

The tumor-bearing mice were randomly divided into 5 groups (A, B, C, D, E), with 5 mice in each group. The mice in different groups were treated as follows: A: control group without any treatment; B: ACG group with intratumoral injection with 50 μL of ACG hydrogel; C: L group with 808 nm laser irradiation (2 W/cm^2^, 10 min); D: ACG (0 h) + L group with intratumoral injection of 50 μL ACG hydrogel followed by immediate 808 nm laser irradiation (2 W/cm^2^, 10 min); E: ACG (12 h) + L group with intratumoral injection of 50 μL ACG hydrogel followed by 808 nm laser irradiation (2 W/cm^2^, 10 min) at 12 h post-injection. The temperature at the tumor site in groups C, D, and E was recorded by an infrared thermometer camera during laser irradiation. Body weights, tumor sizes, and digital photos of tumor-bearing mice with different treatments were recorded for 15 days. The tumor masses in each group were dissected, weighed, and photographed on the 15th day. A typical tumor mass in each group was also immersed with 4% paraformaldehyde for 48 h and then proceeded with an H&E staining procedure to analyze the pathological feature of the tumor.

### 4.11. In Vivo Toxicity Assessment of ACG Hydrogel

One typical mouse in each group of in vivo PTT experiments was sacrificed. Their heart, liver, spleen, lung, and kidney were collected for the H&E staining procedure to study pathologic changes of the main organs to investigate the possible toxicity of ACG hydrogel-based PTT. To further explore the short-term and long-term toxicity of ACG hydrogel in vivo, the liver function and kidney function of the tumor-bearing mice treated with ACG hydrogel were evaluated. The mice without any treatment were set as control. More specifically, the tumor-bearing mice were intratumorally injected with ACG hydrogel (50 μL). Then, the serum of the mice was collected at 0.5 d and 7 d post-injection to measure the liver and kidney function markers (*n* = 3). The liver function markers include total bilirubin (TBIL), alanine aminotransferase (ALT), aspartate aminotransferase (AST), alkaline phosphatase (ALP), total protein (TP) and albumin (ALB). The kidney function markers include creatinine (CREA), uric acid (UA) and urea (UERA).

### 4.12. Statistical Analysis

The data were presented as the means ± standard deviation (SD), and the statistical difference of the results was evaluated using IBM SPSS Statistics 26. For data under normal distribution, differences between multiple groups were analyzed for significance using two-way analysis of variance (ANOVA) post pairwise comparisons with Bonferroni correction or one-way ANOVA followed by LSD post hoc multiple comparisons test, while the data not complying with the normal distribution were evaluated by a nonparametric Kruskal–Wallis test with Bonferroni correction. The threshold of significance was defined as * *p* < 0.05, ** *p* < 0.01, *** *p* < 0.001, respectively.

## Data Availability

Not applicable.

## Supplementary Materials

The following supporting information can be downloaded at: https://www.mdpi.com/article/10.3390/gels8110754/s1, Figure S1: Optimization for the fabrication of ALG–Ca hydrogel; Figure S2: Optimization for the fabrication of ACG hydrogel; Figure S3: The comparation of the dispersive capacity of ACG hydrogel and GP solution (20 mg GP/mL); Figure S4: Dynamic oscillatory time sweep measurements; Figure S5: Shear strain tests; Figure S6: Fluorescence emission spectra of ACG hydrogel and ACG hydrogel mixed with BSA (1 mg/mL); Figure S7: MTT assay of 4T1 tumor cells after incubation with ACG hydrogel; Figure S8: Representative hematoxylin and eosin (H&E) stained tumor tissue sections in photothermal therapy experiments in vivo; Figure S9: The average body weight of the mice.
