# Supplementary material for: In Situ Biosynthesis of Photothermal Parasite for Fluorescence Imaging-Guided Photothermal Therapy of Tumors
Source: Gels. 2022 Nov 21;8(11):754. doi: 10.3390/gels8110754 (PMC9689737; doi:10.3390/gels8110754)
Supplement: Supplementary file 1 [file gels-08-00754-s001.zip › gels-2011038-supplementary.pdf]

In Situ Biosynthesis of Photothermal Parasite for Fluorescence Imaging-Guided Photothermal Therapy of Tumors

Yaqiong Wang [1], Haiyan Pan [2], Zhaowei Meng [3,*] and Cai Zhang [4,*]

[1] Department of Radiology, Zhongshan Hospital, Fudan University, Shanghai 200032, China
[2] Department of Radiology, Tianjin Medical University General Hospital, Tianjin 300052, China
[3] Department of Nuclear Medicine, Tianjin Medical University General Hospital, Tianjin 300052, China
[4] Department of Radiology, Tianjin Medical University Cancer Institute and Hospital, Tianjin 300060, China
* Correspondence: zmeng@tmu.edu.cn (Z.M.); caizhang@tmu.edu.cn (C.Z.)

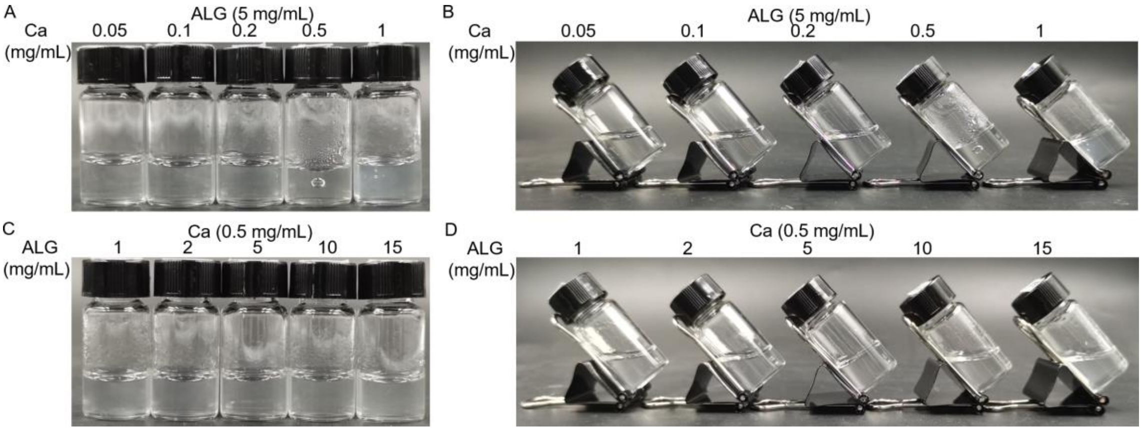

Figure S1. Optimization for the fabrication of ALG-Ca hydrogel. Vertically (A) and obliquely (B) placed mixture of ALG solution (5 mg/mL) and $CaCl_2$ solution (from left to right: 0.05, 0.1, 0.2, 0.5, and 1 mg/mL). Vertically (C) and obliquely (D) placed mixture of $CaCl_2$ solution (0.5 mg/mL) and ALG solution (from left to right: 1, 2, 5, 10, and 15 mg/mL).

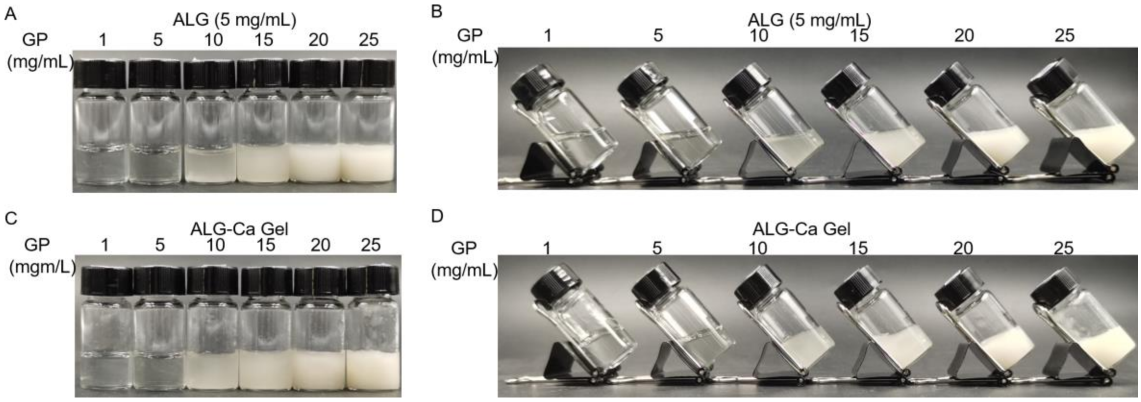

Figure S2. Optimization for the fabrication of ACG hydrogel. Vertically (A) and obliquely (B) placed mixture of ALG solution (5 mg/mL) and GP solution (from left to right: 1, 5, 10, 15, 20, and 25 mg/mL). Vertically (C) and obliquely (D) placed mixture of ALG-Ca hydrogel (5 mg/mL ALG and 0.5 mg/mL $CaCl_2$) and GP solution (from left to right: 1, 5, 10, 15, 20, and 25 mg/mL).

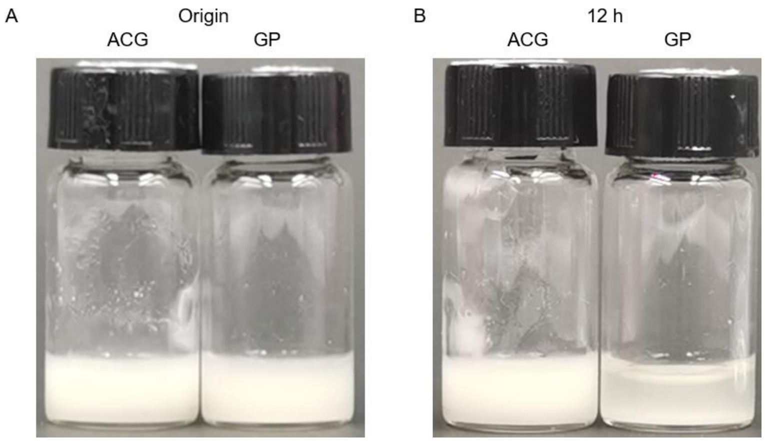

Figure S3. The comparation of the dispersive capacity of ACG hydrogel and GP solution (20 mg GP/mL). (A) The original state of ACG hydrogel and GP solution. (B) The state of ACG hydrogel and GP solution after 12 h of immobility.

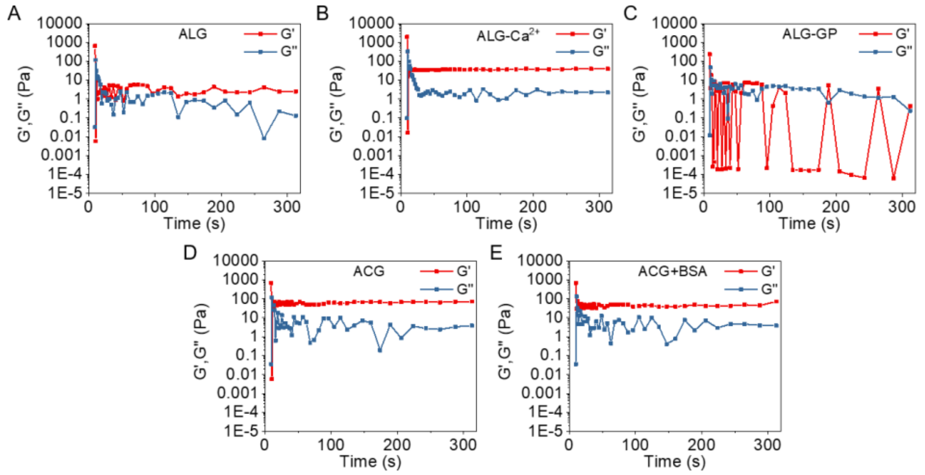

Figure S4. Dynamic oscillatory time sweep measurements of ALG solution (A), ALG-Ca^{2+} hydrogel (B), ALG-GP solution (C), ACG hydrogel (D) and ACG+BSA hydrogel (E) with a strain amplitude of 1% and an angular frequency of 1 rad/s. G': storage modulus, G": loss modulus.

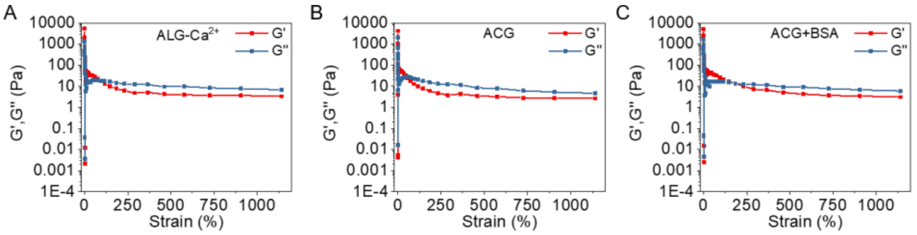

Figure S5. Shear strain tests of ALG-Ca^{2+} hydrogels (A), ACG hydrogels(B) and ACG+BSA hydrogels (C) with a shear rate from 0.1% to 1140%. G': storage modulus, G'': loss modulus.

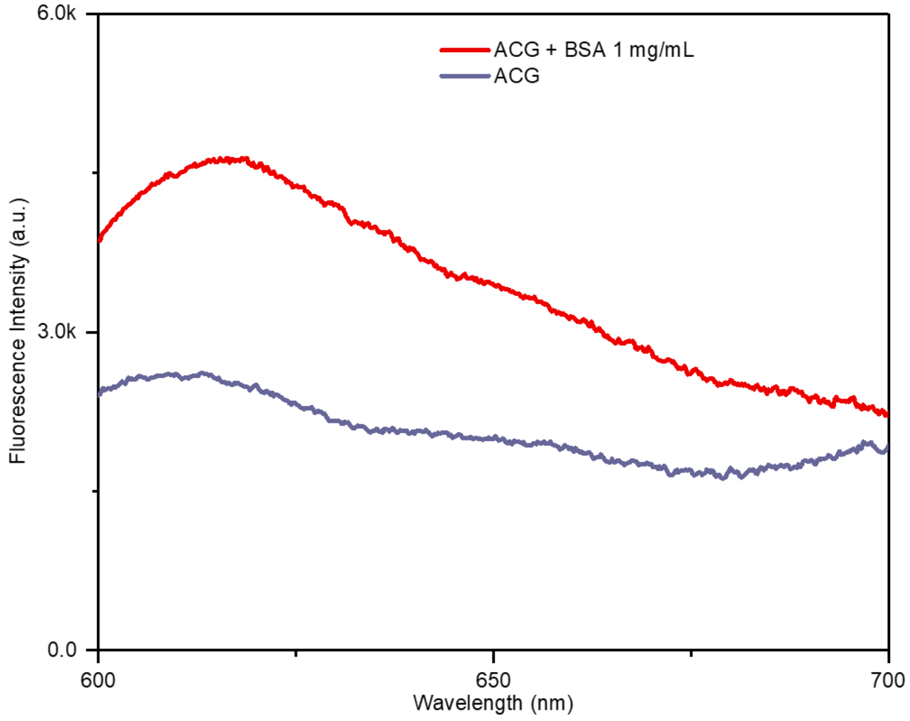

Figure S6. Fluorescence emission spectra of ACG hydrogel and ACG hydrogel mixed with BSA (1 mg/mL).

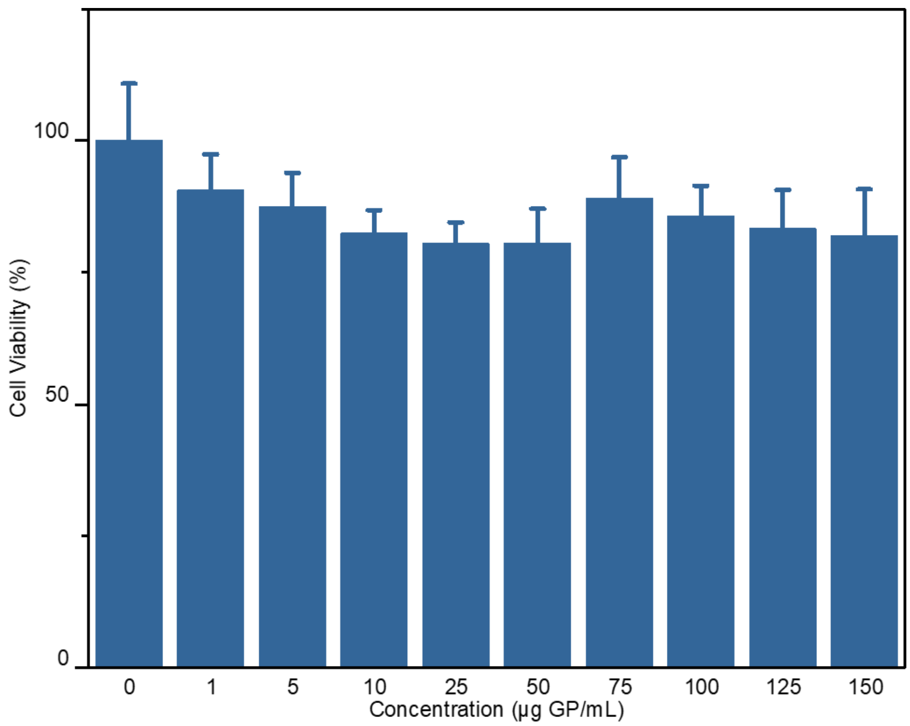

Figure S7. MTT assay of 4T1 tumor cells after incubation with ACG hydrogel (0 to 150 μg GP/mL), shown as means ± SD, n = 6.

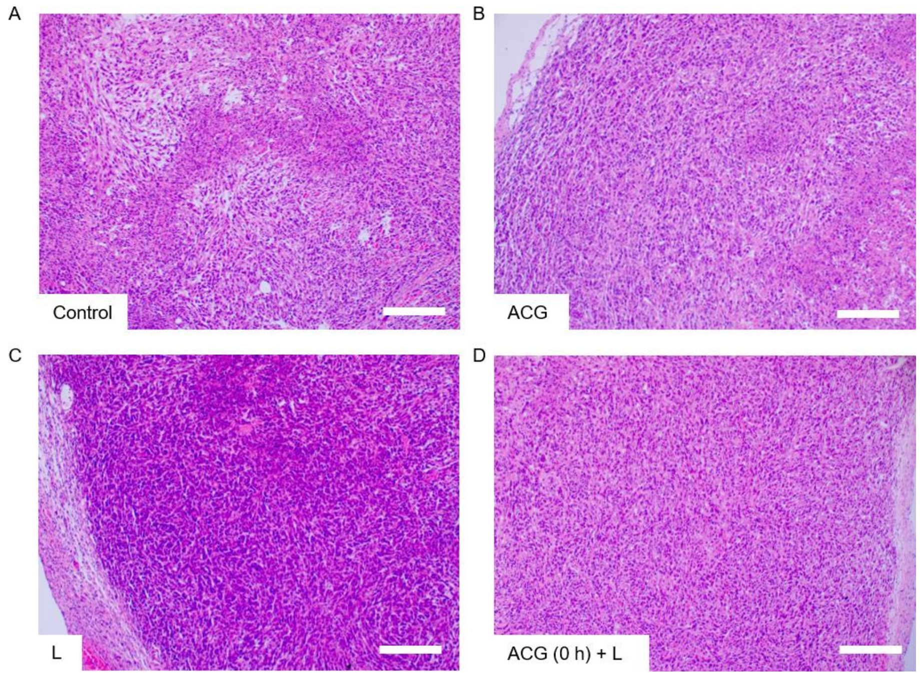

Figure S8. Representative hematoxylin and eosin (H&E) stained tumor tissue sections in photothermal therapy experiments *in vivo*. (A) Control group. (B) ACG group. (C) L group. (D) ACG (0 h) + L group. Scale bar: 200 μm. ACG: intratumoral injection of 50-μL ACG hydrogel. L: 808 nm laser irradiation (2 W/cm^2, 10 min). ACG (0 h) + L: intratumoral injection of 50-μL ACG hydrogel followed by immediate laser irradiation (2 W/cm^2, 10 min).

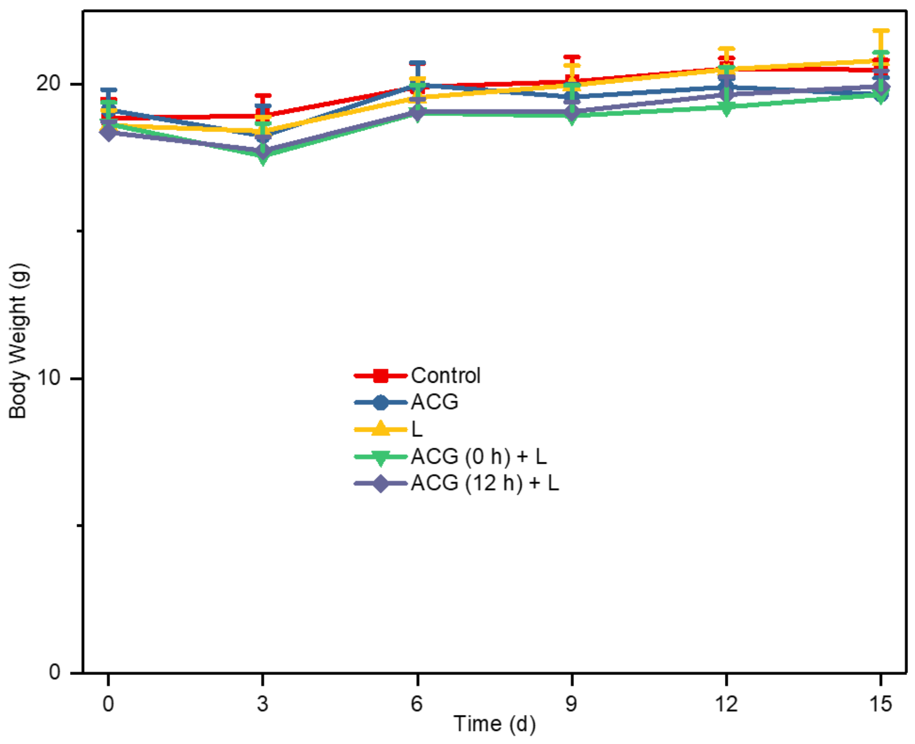

Figure S9. The average body weight of the mice, shown as means ± SD, n = 5. ACG: intratumoral injection of 50-μL ACG hydrogel. L: 808 nm laser irradiation (2 W/cm^2, 10 min). ACG (0 h) + L: intratumoral injection of 50-μL ACG hydrogel followed by immediate laser irradiation (2 W/cm^2, 10 min). ACG (12 h) + L: intratumoral injection of 50-μL ACG hydrogel followed by 12-h delayed laser irradiation (2 W/cm^2, 10 min).
